# Autoimmune regulation of chronic pain

**DOI:** 10.1097/PR9.0000000000000905

**Published:** 2021-03-09

**Authors:** Michael J. Lacagnina, Cobi J. Heijnen, Linda R. Watkins, Peter M. Grace

**Affiliations:** a Laboratories of Neuroimmunology, Department of Symptom Research, University of Texas MD Anderson Cancer Center, Houston, TX, USA; b Department of Psychology and Neuroscience, Center for Neuroscience, University of Colorado, Boulder, CO, USA

**Keywords:** Neuropathic pain, Inflammatory pain, Neuroinflammation, Neuroimmune, Glia

## Abstract

Chronic pain is an unpleasant and debilitating condition that is often poorly managed by existing therapeutics. Reciprocal interactions between the nervous system and the immune system have been recognized as playing an essential role in the initiation and maintenance of pain. In this review, we discuss how neuroimmune signaling can contribute to peripheral and central sensitization and promote chronic pain through various autoimmune mechanisms. These pathogenic autoimmune mechanisms involve the production and release of autoreactive antibodies from B cells. Autoantibodies—ie, antibodies that recognize self-antigens—have been identified as potential molecules that can modulate the function of nociceptive neurons and thereby induce persistent pain. Autoantibodies can influence neuronal excitability by activating the complement pathway; by directly signaling at sensory neurons expressing Fc gamma receptors, the receptors for the Fc fragment of immunoglobulin G immune complexes; or by binding and disrupting ion channels expressed by nociceptors. Using examples primarily from rheumatoid arthritis, complex regional pain syndrome, and channelopathies from potassium channel complex autoimmunity, we suggest that autoantibody signaling at the central nervous system has therapeutic implications for designing novel disease-modifying treatments for chronic pain.

## Abstract 2

Autoantibodies can contribute to peripheral and central sensitization through activation of complement pathway, activation of neuronal Fc gamma receptors, and disrupted function of neuronal ion channels.

## 1. Introduction

Chronic pain is a serious health condition that dramatically reduces quality of life. Managing the symptoms of chronic pain has become a pervasive problem. From 2015 to 2018, 10.7% of all adults in the United States used prescription pain medication to manage their symptoms.^63^ Existing medications for pain are often only partially effective and can have their own devastating side effects, as exemplified by the dramatic rise in opioid-related overdoses.^38,148^ Despite wide recognition of these issues, there has been little progress in developing improved therapeutics for chronic pain.^59^ A fundamental issue facing researchers has been a knowledge gap in understanding the mechanisms that establish chronic pain and prevent its resolution.^134^ In addition to alterations in neuronal functioning, evidence now implicates bidirectional communication with the immune system in the initiation and maintenance of chronic pain.^57,113^ Clarifying how neurons and immune cells directly influence each other could allow for rational therapeutic design to relieve chronic pain and inappropriate inflammation.^9^

The immune system works in tandem with the somatosensory nervous system to coordinate host defense. Dysregulation of homeostatic reciprocal signaling between nociceptors and immune cells can lead to heightened excitation of sensory neurons and induce pain.^18,48,57^ The underlying mechanisms of neuromodulation and dysfunctional synaptic plasticity from neuroimmune signaling—principally mediated through cytokines, reactive oxygen and nitrogen species, growth factors, and bioactive lipids—have been described elsewhere in detail.^19,57,74,86,113,152^

Excessive inflammation and/or tissue damage can lead to autoimmune responses, characterized by activation of T-cell clones that recognize self-antigens.^40^ Although autoimmune diseases are differentiated by disease-specific immune responses, pathological pain is a shared symptom for most autoimmune diseases.^116^ Although autoimmune signaling has received less attention than other forms of neuroinflammatory signaling, there is now a growing appreciation that autoimmune mechanisms may be a conduit for the development of chronic pain.^27^

In the following review, we will discuss how the immune system can contribute to chronic pain through a variety of specific autoimmune mechanisms. Loss of immunological tolerance (ie, the breakdown or failure of the usual unresponsiveness of the immune system to self-antigens) can occur when autoreactive T-cell clones expand and induce antibody production by autoantigen-specific B cells.^112,143^ The evidence supporting the role of T cells as contributing to both the onset and resolution of chronic pain has been recently reviewed.^89^ Here, we will instead focus on the emerging evidence for the role of autoantibodies (ie, antibodies directed against self-antigens) as actively participating in states of persistent pain through their interactions with the complement system, immune receptors, and neuronal self-antigens.^28,49,186^

**Figure 1.**
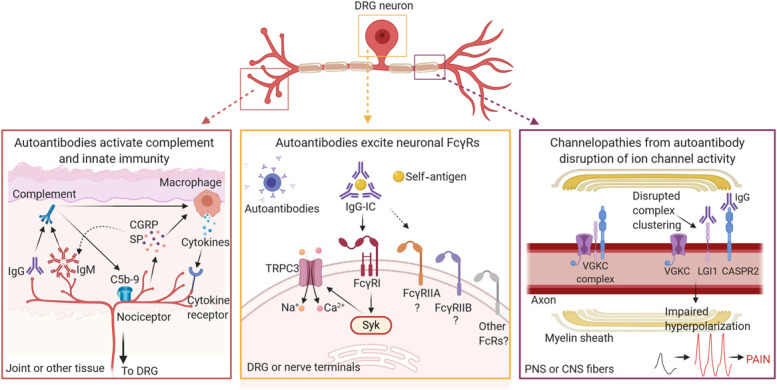
Autoimmune mechanisms influencing nociceptor hyperexcitability leading to chronic pain. Autoantibodies can cause nociceptor hyperexcitability and pain through multiple mechanisms across the neuraxis. In tissues such as the joint, accumulation of IgG and/or IgM autoantibodies can activate the complement system. Activation of subsequent complement proteins promotes inflammation and cytokine release from recruited macrophages (or other immune cells) and leads to tissue damage through accumulation of pore-forming membrane attack complex (C5b-9). Feedback from peripheral nociceptors also influences local tissue immunity by releasing neurotransmitters or peptides, such as CGRP or SP, to recruit or modulate the activity of immune cells, or to influence deposition of IgM. At the DRG cell body and its terminals, IgG-ICs bound to self-antigen activate FcγRs expressed on neurons. FcγRI can signal through Syk, causing release of intracellular stores of calcium that trigger opening of TRPC3 channels, depolarizing the cell leading to increased firing. Neuronal FcγRI activity is known to activate sensory neurons, yet it remains unknown if other classes of FcγRs are important for pain. At myelin sheaths on axons, IgG autoantibodies that target components of the VGKC complex disrupt appropriate channel clustering at juxtaparanodal regions, disturbing normal hyperpolarizing currents and leading to increased action potential firing. Collectively, the outcome of these autoantibody interactions is increased neuronal hyperexcitability, leading to pain. CASPR2, contactin-associated protein-like 2; CGRP; calcitonin gene-related peptide; CNS, central nervous system; C5b-9, complement 5b-9 membrane attack complex; DRG, dorsal root ganglia; FcγR, Fc gamma receptor; IgG, immunoglobulin G; IgG-IC, immunoglobulin G immune complex; IgM, immunoglobulin M; LGI1, leucine-rich glioma inactivated 1; PNS, peripheral nervous system; SP, substance P; SYK, spleen tyrosine kinase; TRPC3, transient receptor potential canonical 3; VGKC, voltage-gated potassium channel.

The framework of this review follows the sequence of events for an autoimmune reaction and examines evidence at each step for its contribution to pain. These events include (1) the recognition of self-antigen by B cells; (2) the production of autoantibodies by B cells, focusing on immunoglobulin G (IgG) and immunoglobulin M (IgM); (3) activation of complement; (4) autoantibody signaling at antibody receptors (Fc receptors); and (5) direct interactions of autoantibodies at neuronal antigens (Fig. 1). These key cellular and molecular participants are described in the context of several disease examples—including autoimmune disease, traumatic injury, and channelopathies—in which chronic pain can be attributed to autoimmune mechanisms.

## 2. B cells and their role in chronic pain

### 2.1. B cells: overview

**Figure 2.**
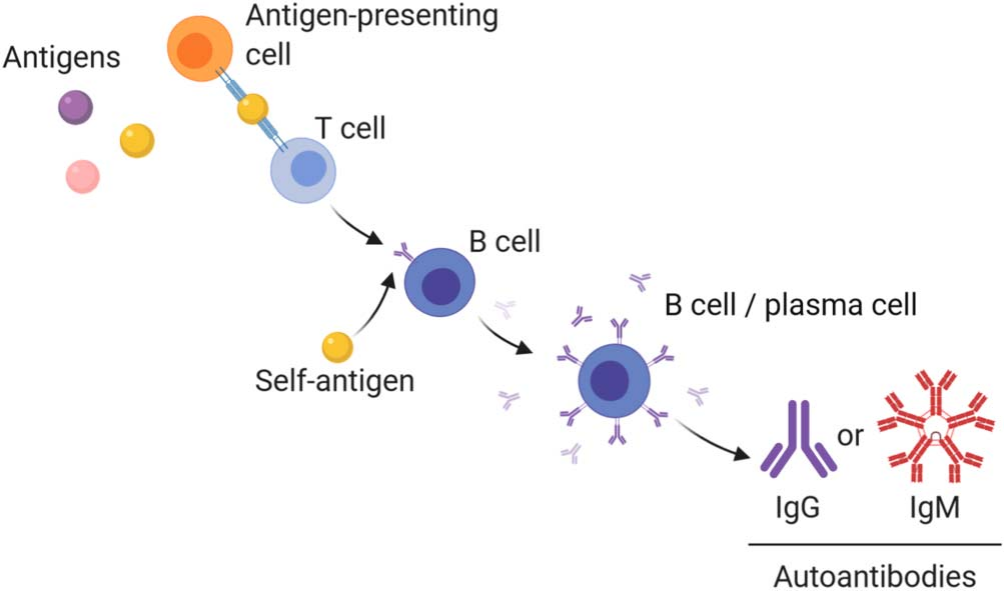
B-cell development and production of autoantibodies. Autoimmunity develops when a self-antigen breaks immunological tolerance. This process is believed to occur when antigen-presenting cells deliver self-antigen to naive T cells expressing self-reactive receptors, causing maturation into different T-cell classes: either CD8^+^ (cytotoxic or suppressor T cells) or CD4^+^ (helper T cells). Autoreactive helper T-cells clones then present self-antigen to autoreactive B cells; alternatively, some antigens may activate B cells without the help of T cells. On receptor recognition and in the presence of co-stimulatory signals, the B cell will differentiate into a plasma cell and begin secreting antibodies that recognize the self-antigen. In the context of pain, there is evidence that both IgG and IgM can influence nociceptor activity. This can occur by binding to their antigen, activating the complement system, or signaling at their respective Fc receptors. IgG, immunoglobulin G; IgM, immunoglobulin M.

The adaptive immune system uses B cells to achieve long-lasting and selective defense against pathogens through the production and secretion of antibodies.^85^ Originating from fetal liver and adult bone marrow, immature B cells undergo differentiation at germinal centers in secondary lymphoid tissues, such as the spleen or lymph nodes.^88,90^ During a typical immune response, CD4^+^ helper T cells first recognize antigen presented by major histocompatibility complex (MHC) class II molecules from antigen-presenting cells through their surface-bound T-cell receptors.^37,89^ On encountering foreign or host antigen presented by helper T cells and recognized by cognate B-cell receptors, B cells become activated and undergo further proliferation and maturation either into memory B cells or antibody secreting cells, both short-lived plasmablasts and long-lived plasma cells.^85,88,90,123^ B-cell activation and differentiation can also occur through T-cell–independent pathways and occur outside of germinal centers.^85^ During B-cell maturation, there are several checkpoints to eliminate B cells that recognize self-antigen through receptor editing, negative selection, or anergy, which occur throughout the developmental lifespan of B cells.^46,120^ However, if these checkpoints are disrupted, self-reactive B cells can escape removal and differentiate into plasmablasts or plasma cells that produce autoantibodies (Fig. 2).^164^ For this reason, some current therapeutics for treating autoimmune diseases either target immune functions of B cells or eliminate B-cell populations entirely.^40^

### 2.2. B cells and pain

There is some clinical evidence that B cells play a relevant role in the pathogenesis of rheumatoid arthritis (Box 1). Depletion of B cells can be achieved therapeutically with rituximab, a monoclonal antibody that targets CD20 expressed by most B cells and leads to cell death through complement-dependent cytotoxicity.^147^ In randomized controlled trials, infusions of rituximab to deplete B cells can significantly improve disease symptoms, including pain.^34^ Likewise, the necessity for these cells in developing rheumatoid arthritis symptoms can be tested in mice that lack functioning B cells. Transgenic μMT mice with no functioning B cells are fully resistant to developing arthritis in the collagen-induced arthritis model, although pain-related behaviors were not specifically measured.^160^

Text box 1. **Rheumatoid arthritis**

Rheumatoid arthritis is a chronic autoimmune disease that is distinguished by joint inflammation of the synovial membrane, erosion of bone and cartilage, and joint pain (arthralgia). During disease progression, the synovium is typically infiltrated by innate immune cells (including macrophages, dendritic cells, neutrophils, and mast cells) that release proinflammatory cytokines and chemokines, which recruit additional cells of adaptive immunity (including T cells and B cells).^82^ The interaction of these secreted immune mediators with fibroblast-like synoviocytes and neighboring immune cells establishes an environment leading to arthralgia, tissue destruction, and bone erosion.^82,84,187^ Our understanding of disease progression in arthritis has been greatly facilitated by several different animal models, which usually involves immunization against collagen antigens, transfer of anticollagen antibodies or serum, or spontaneous genetic mouse strains.^82^

Most patients with rheumatoid arthritis indicate pain is the symptom they would most like to improve.^66^ Release of cytokines, such as tumor necrosis factor, interleukin-1β, interleukin-6, interferon gamma, and other proinflammatory mediators, from cells near the inflamed joint may act to directly activate peripheral nociceptors, and there is evidence that plasticity in both peripheral and central pain processing may further contribute to aberrant pain.^55,106^ Patients can experience pain localized to the joints, as well as neuropathic symptoms, such as burning, electrifying sensations, or pain to light pressure and dynamic brushing. These symptoms further suggest that alterations in peripheral and central processing occur in rheumatoid arthritis, and these mechanisms may share some similarities with neuropathic pain conditions.^81,177^

There is accumulating evidence that pain in rheumatoid arthritis may be maintained independently from ongoing inflammation. Joint pain frequently occurs before diagnosis and before signs of inflammation are present.^91^ Even when edema and inflammation of the joints are relieved by disease-modifying antirheumatic drugs, pain can persist in a substantial portion of patients.^114,115,162^ Beyond joint pain, patients with rheumatic diseases also report higher incidences of both migraine and neuropathic pain despite clinical control of inflammation-related symptoms.^111^ Identifying how pain arises in rheumatoid arthritis separate from ongoing inflammation could provide new avenues for clinically relevant pain relief.

A role for B cells has also been demonstrated in the tibial fracture and casting model, a rodent model of complex regional pain syndrome (CRPS) (Box 2). Depletion of B cells with intravenous anti-CD20 antibody (a murine analog of rituximab) 1 week after tibial fracture reduces the severity of mechanical allodynia and vascular changes to the injured paw after cast removal.^98^ Delayed treatment of anti-CD20 was also successful at attenuating the severity of mechanical allodynia and weight bearing on the injured paw, indicating both development and maintenance of CRPS-like nociceptive symptoms involve B cells.^98^ Similar attenuation of fracture-induced allodynia and edema was observed in transgenic B-cell-deficient μMT mice.^98^ These preclinical results demonstrate a likely role for B cells in CRPS-associated pain, although this hypothesis has yet to be clinically tested.

Text box 2. **Complex regional pain syndrome**

Complex regional pain syndrome (CRPS) is a debilitating form of posttraumatic pain. The etiology of CRPS is unclear, but typically emerges after injury or surgery of a limb. Symptoms between patients are highly heterogenous but can include sensory abnormalities (chronic pain of the affected limb, including spontaneous pain and allodynia or hyperalgesia), motor dysfunctions (loss of motor coordination and dystonia), autonomic dysfunctions (vasomotor instability, skin color change and warming skin temperature), and trophic changes (increased or decreased hair and nail growth and tissue or bone dystrophy).^125,186^ In addition to the inciting trauma, immobilization of the injured limb (such as wearing a cast) is a risk factor for developing CRPS.^3,165^

The complexity of the clinical manifestation has made it difficult for researchers to understand the mechanistic underpinnings of this syndrome. Heterogeneity of the clinical presentation signifies a likely multisystem pathology involving inflammatory, neuronal, musculoskeletal, and microvascular contributions that may vary over disease progression.^13,44^ Evidence from patients with CRPS and animal models of the disease points to immune cell activation, especially in the acute phase of the disorder.^92,181^ Elevated concentration of tumor necrosis factor, interleukin-1β (IL-1β), IL-6, IL-8, soluble tumor necrosis factor receptors, monocyte chemoattractant protein-1, macrophage inflammatory protein-1β, soluble receptor for advanced glycation end products, and other neuroimmune signaling factors were detectable in patients skin, blood, or serum.^131^ Biopsies of the affected skin reveal keratinocyte activation, mast cell accumulation and degranulation, and increased number of tissue-resident Langerhans cells, although both mast cell and Langerhans cell numbers may resolve in advanced stages of the disease.^14,130^ Mass cytometry performed on blood from patients with CRPS reveals an expanded population of T-lymphocyte subpopulations, including memory CD4^+^ and CD8^+^ T cells, suggesting an ongoing inflammatory lymphocyte response in CRPS.^149^ Activation of spinal cord glial cells may also play a role in altering central processing of nociceptive signals.^2^ Noninvasive positron emission tomography imaging of translocator protein-18 kDa (TSPO) to monitor microglia and myeloid cells reveals early and persistent myeloid cell activation in the tibial fracture/casting mouse model, along with transient activation of spinal cord and brain microglia.^24^ Understanding the temporal dynamics of immune cell activation along the pain neuraxis in CRPS may offer new insights into disease progression.

Depletion of B cells may also be a disease-modifying treatment for pain in voltage-gated potassium channel (VGKC) complex autoimmunity (Box 3 and Fig. 1, right panel), although the existing evidence is only from small sample sizes. In a small patient sample with anti-VGKC complex antibodies, rituximab treatment showed reversal of clinical symptoms in some patients.^71^ A case study with intractable Morvan syndrome and anti-VGKC complex antibodies also described that pain relief for the patient was achieved with rituximab treatment.^128^

Text box 3. **Potassium channel complex autoimmunity**

There are several neurological conditions that are characterized by the presence of autoantibodies that target components of the voltage-gated potassium channel complex, including acquired neuromyotonia, Morvan syndrome, and autoimmune limbic encephalitis.^72^ The clinical features of these neuronal autoimmune disorders involve peripheral nerve hyperexcitability and include spontaneous muscle twitching, muscle cramps or stiffness, dysautonomia, epilepsy, insomnia, and neuropathic pain.^65,117^ In patients with confirmed autoantibodies against voltage-gated potassium channel complexes, around half suffer from some form of chronic pain, and 28% of patients report pain as their only symptom.^79^

### 2.3. Future directions for B cells and their role in chronic pain

There is still much more research needed to determine the extent to which B cells contribute to pain. B-cell–deficient mice have been shown to develop typical mechanical allodynia to the spared nerve injury model of neuropathic pain.^21^ These data suggest that the contribution of B cells to pain-related behavior may be dissimilar between different painful conditions. A key limitation to these studies is that these B-cell manipulations are systemic, either using genetically deficient transgenic animals or through pharmacological depletion. Drugs such as rituximab can deplete CD20^+^ B cells that differentiate into short-lived autoantibody-producing plasmablasts, but this intervention may not eliminate terminally differentiated long-term plasma cells, making it harder to draw strong conclusions from these manipulations alone.^151^ As a result, an unanswered question is whether there are distinct subpopulations of B cells that are pathogenic and chiefly contribute to pain, which may depend on the subclass of antibodies produced and the avidity/affinity for their cognate antibody receptor. It is conceivable that there are specific tissue niches that support unique B-cell populations, and the release of cytokines or autoantibodies from these pathogenic B cells may interact with nociceptors at nerve terminals (Fig. 1, left panel), at nociceptor cell bodies in the dorsal root ganglia (DRG) (Fig. 1, center panel), or at cells in the spinal cord by entry through blood or lymphatic vessels.^73^

New technological advancements in single-cell sequencing and highly multiplexed profiling of cell identity could shed light on these questions. For instance, a recent high-dimensional profiling of human B cells identified unique populations of tissue-resident B cells, which may have tissue-specific functions related to antibody release, plasma cell differentiation, toll-like receptor expression, or other functional properties.^47^ Furthermore, as noted in Box 1, in rheumatoid arthritis the joint synovium is populated by multiple inflammatory cell types that contribute to joint damage and disease progression, including unique populations of B cells. Combining transcriptional sequencing with mass cytometry revealed distinct expanded cell populations in synovial tissue associated with rheumatoid arthritis, including autoimmune-associated *ITGAX*^*+*^ *TBC21*^*+*^ B cells in samples with high leukocyte infiltration, along with transcriptionally distinct CD8^+^ and CD4^+^ T cells.^188^ These high-dimensional data sets highlight how inflammatory cross-talk between local and infiltrating cells within the tissue microenvironment can recruit or establish unique immune cell identities. In the context of chronic pain disorders, it remains to be investigated if localized activation of B cells occurs within specific niches of the pain neuraxis.

## 3. Autoantibodies and their role in chronic pain

Recent advancements in immunology and neuroscience have elucidated several effector functions of autoantibodies in the pathogenesis of different chronic pain conditions. Pain in autoimmune disease has typically been attributed to localized or systemic inflammation resulting from antibody-mediated complement activation or by stimulating release of proinflammatory cytokines or peptides. However, chronic pain in autoimmune disease can be present before cardinal signs of inflammation are evident (such as in rheumatoid arthritis), or in some circumstances, pain may be the only symptom of a diagnosed autoantibody disorder (such as in VGKC complex autoimmunity).^79,114^ Here and in subsequent sections, we will focus on evidence supporting the emerging view that autoantibodies may engage different mechanisms of action that can promote chronic pain.

### 3.1. Autoantibodies: overview

Antibodies (Ig for immunoglobulin) are Y-shaped proteins segregated into 2 domains: the variable antigen-binding domain (Fab region) at the top that can recognize unique epitopes on antigens and the invariant fragment crystallizable domain (Fc region) at the bottom, which enables the antibody to interact with Fc receptors and elements of the complement system.^153^ There are 5 classes of Ig in mammals: IgA, IgD, IgE, IgG, and IgM, each with different structures and functions for host immunity. To date, IgG and IgM autoantibodies in particular have been implicated in persistent pain states.^20,49,62,186^

On binding antigen, IgM or IgG function to neutralize targets by agglutination, targeting cells for opsonization or antibody-dependent cell cytotoxicity, and recruiting elements of the complement cascade. Antibodies can also form immune complexes—clusters of multiple antibodies and antigens bound together—and these complexes can signal at their respective Fc receptors, either Fc mu receptor (FcµR) for IgM or Fc gamma receptor (FcγR) for IgG.^153^ Antibodies recognize epitopes from pathogens but are also important for wound healing and removal of cellular debris after injury because both IgM and IgG accumulate at the site of sciatic nerve crush injury and contribute to clearance of myelin debris.^173^ Autoantigenic antibodies in autoimmune diseases are most frequently of the IgG isotype, although other Ig classes can also promote autoimmunity.^46^

Although considerable advancements have been made toward understanding the role of autoantibodies across diseases, for the most part it is still unclear how initial tolerance is broken leading to B-cell expansion, autoantibody production, and epitope spreading (ie, antibody reactivity to epitopes distinct from the epitope that initiated the immune response).^151^ One possibility identified in rheumatoid arthritis for generating self-reactive antibodies is through posttranslational modifications of proteins. Posttranslational modifications—such as citrullination, glycosylation, carbamylation (or homocitrullination), acetylation, phosphorylation, nitrosylation, and sulfation—alter the chemical, functional, and antigenic properties of naturally occurring proteins or peptides in various ways, including the addition or removal of functional groups, sugars, or other chemical groups to particular sites on proteins.^42,175^ These modifications to protein structures or amino acid sequences can generate new antigenic epitopes capable of producing a CD4^+^ T-cell- and MHC class II-dependent autoantibody response against native protein that breaks immunological tolerance.^42^

### 3.2. Autoantibodies and pain in rheumatoid arthritis

Autoantibodies have been suggested as a potential mechanism driving pain in rheumatoid arthritis.^17^ Although the etiology of the disease is unknown, autoantibodies against cyclic citrullinated peptide and rheumatoid factor can be detected in blood several years before diagnosis.^139,140^ Anti-citrullinated protein antibodies (ACPAs) can interact with proteins or peptides expressing the amino acid citrulline.^43^ The generation of autoantigenic proteins expressing citrulline occurs through the process of citrullination, a posttranslational modification in which the amino acid arginine is replaced by citrulline through the enzymatic activity of peptidylarginine deiminases (PADs).^26^ Peptidylarginine deiminase enzymes have been implicated in generating modified citrullinated proteins (such as fibrinogen, vimentin, collagen type II, fibronectin, histones, and α-enolase) that are targeted by ACPAs, and PADs may themselves become targets of autoantibodies during disease progression.^15,26^ Anti-citrullinated protein antibody–IgG is reduced but not completely eliminated by rituximab, indicating that ACPAs are produced by CD20^+^ B cells, with a persisting contribution by stable, terminally differentiated plasma cells.^151^ The observation that both joint pain and the presence of autoantibodies occur before clinical onset of arthritis suggests that autoimmune antibody signaling could be implicated as a distinct mechanism for arthralgia.

Anti-citrullinated protein antibodies are a biomarker of rheumatoid arthritis and may independently promote pain-related behavior. Intravenously administered IgG isolated from ACPA seropositive patients is sufficient to produce mechanical and thermal hypersensitivity in mice, along with nonevoked reductions in spontaneous activity without producing joint inflammation.^183^ These results indicate that anti-citrullinated antibodies from patients with rheumatoid arthritis are sufficient to promote pain. Anti-citrullinated protein antibodies accumulate in disease-relevant tissues including the joints, skin, bone marrow, DRG, adipose tissue, and spleen. Patient-derived ACPAs localize with CD68^+^ macrophages or osteoclasts, alongside CGRP^+^ neurons in the bone marrow, and its effects on pain was related to increased expression of the chemokine interleukin-8 (IL-8).^183^ Anti-citrullinated protein antibody stimulation of IL-8 could contribute to arthralgia through direct action of IL-8 at its receptor cysteine-x-cysteine receptor1/2 on small fiber nociceptors, thereby increasing voltage-gated sodium currents, or indirectly through recruitment of neutrophils.^16,178^ Likewise, when monoclonal antibodies are cloned from the sequences of citrulline-autoreactive B-cell receptors captured from ACPA^+^ patients, some (but not all) patient-derived antibodies can produce mechanical hypersensitivity in mice after injection of lipopolysaccharide (LPS) to model an inflammatory stimulus.^166^ Thus, the mechanism for how ACPAs produce pain may require an additional inflammatory event and result from the formation of local immune complexes within the joints.^43^

Beyond ACPAs, autoantibodies in the collagen antibody-induced arthritis (CAIA) preclinical model are also pronociceptive. Using the CAIA model, antibodies targeting collagen type II (anti-CII) are transferred to animals, followed by LPS injection to trigger an inflammatory response. Mice treated with anti-CII antibodies develop mechanical hypersensitivity and show reduced spontaneous activity several days before any signs of inflammation or extracellular remodeling are observed in the joints.^12^ Fab fragments of these anti-CII antibodies are ineffective at producing pain-related behavior; these data indicate that the Fc fragment of these anti-CII autoantibodies may be necessary for their effects on pain, and further research is needed to determine if the Fc fragment is sufficient to account for pain in this model.^12^ When applied to primary DRG cell cultures, immune complexes of anti-CII IgG bound to collagen type II stimulate intracellular calcium release and evoke inward currents in around 20% of cells (and 42% of likely transient receptor potential V1^+^ capsaicin-sensitive cells), whereas monomeric antibodies that are not in immune complexes fail to evoke any DRG activity.^12^ This may explain why treatment of cultured DRGs with patients' ACPA does not on its own stimulate depolarizing currents becasue formation of immune complexes (self-antigen bound to autoantibodies) is likely necessary for autoantibodies to cause neuronal hyperexcitability.^183^

### 3.3. Autoantibodies and pain in complex regional pain syndrome

The development of CRPS comprises elements of sterile inflammation and autoantibody-mediated pathology, leading some to argue that CRPS should be considered as an autoimmune syndrome.^20,52^ Early evidence for an autoantibody hypothesis of CRPS was derived from experiments in which intravenous immunoglobulin (IVIg) treatment (pooled human serum consisting mainly of IgG) resulted in pain relief for small samples of patients with CRPS.^50,53^ In a larger multicenter trial, however, IVIg was not more effective than placebo for relieving pain, suggesting that this method may only be efficacious for a certain subpopulation of patients.^51^ Although this evidence is partially supportive of circulating autoantibodies as contributing to pain in a subset of CRPS, it should be noted that the therapeutic mechanism of action for pooled IVIg is still debated; the salutary effects of IVIg could involve inhibition of antibodies binding to activating FcγRs, upregulation of inhibitory FcγRIIB, neutralization of circulating IgG, or through alternative methods of immunomodulation.^94^ Plasma exchange therapy has also been retrospectively associated with pain relief in a high proportion of patients with long-standing CRPS.^7^ Similar to the caveats for IVIg, plasma exchange might remove alternative disease-relevant molecules or proteins other than pathological autoantibodies, such as miRNA-containing exosomes.^13^

Passive IgG transfer, in which IgG fractions are purified from serum of patients with CRPS or rodent models and delivered to naive subjects, can address if autoantibodies are necessary for pain in CRPS.^27^ Serum IgG from patients with chronic CRPS transferred intraperitoneally to female mice, followed by unilateral plantar skin and muscle incision to model limb injury, enhances unilateral mechanical hyperalgesia and paw edema compared with IgG from healthy matched controls.^67,163^ With daily transfer of IgG from patient with CRPS, mechanical hypersensitivity of the paw quickly develops and remains heightened over time. By contrast, paw edema and concentration of the inflammatory cytokines tumor necrosis factor, IL-1β, and IL-6 in the paw increases at the time of injury but resolves by day 13, when mechanical allodynia is still elevated.^67^ Reminiscent of the passive IgG transfer effects from patients with CRPS, serum or purified IgM from wild-type mice 3 weeks after tibial fracture, as well as IgM purified from patients with CRPS, was pronociceptive when transferred to B-cell–deficient mice.^61,62^ These observations support the notion that injury-induced self-antigens trigger production of autoantibodies that could produce pain through activation of nociceptive neurons.^61^ In agreement with this hypothesis, IgG purified from patients with CRPS and treated on mouse skin–saphenous nerve preparations evokes activity of Aδ and C mechanonociceptors.^25^ The recapitulation of these basic CRPS-like pain symptoms in the rodent model after passive antibody transfer suggests that pathogenic autoantibodies are present in CRPS and directly contribute to pain-related behavior after injury.

The characteristics of the cellular and molecular targets for autoantibodies in CRPS are not fully understood. One possibility is the generation of antibodies that recognize G protein-coupled receptors on neurons or vascular tissue after traumatic injury. Antibodies isolated from patients with CRPS have demonstrated to be agonists of autonomic neuronal receptors, including β2 adrenergic, muscarinic 2, and α-1a adrenergic receptors.^32,80^ Epidermal adrenergic signaling has been associated with pain and inflammation in CRPS; keratinocytes in the epidermis also express β2 adrenergic receptors, and stimulation of these receptors by sympathetic neurons contributes to local release of IL-6 in a rat model of CRPS.^96^ This pathway could indirectly increase cutaneous sensory neuron excitability and promote mechanical hypersensitivity seen in CRPS.^96^ Alternatively, skin-nerve preparations demonstrate that IgG from patients with CRPS may directly stimulate activity from peripheral nociceptors, although additional in vivo evidence of electrophysiological properties is needed to confirm these observations.^25^ Injury-induced changes in neuronal neuropeptide activity may also influence the localized immune response. Transgenic mice with deficient expression of the neuropeptides substance P (*Tac1*^−/−^) or CGRP (*Ramp1*^−/−^) show attenuated mechanical allodynia in the tibial fracture and casting model of CRPS.^97^ In addition, *Ramp1*^−/−^ mice have reduced deposition of IgM in the skin, sciatic nerve, and spinal cord compared with wild-type animals 3 weeks after fracture.^97^ Thus, neuropeptide signaling may influence IgM autoantibody deposition after injury, and the antigenic targets of IgM may be localized to the skin and nerves. Indeed, IgM from the mouse model of CRPS and IgM from patients with CRPS have been shown to be autoreactive to keratin 16 (KRT16) from the epithelial tissues and cross-reactive with histone 3.2, γ-actin, and α-enolase.^62,161^ Immunohistochemical staining indicates microglial and astrocyte activation also occurs in the dorsal horn of the spinal cord after intraperitoneal transfer of IgG from patients with CRPS.^67^ Systemic IL-1 inhibition or microglia-specific elimination of IL-1β alleviates the pain-related behavior from IgG transferred from patients with CRPS, supporting a role for central glial activation in the development of mechanical allodynia after CRPS IgG transfer. Collectively, these data indicate that IgG and IgM autoantibodies in CRPS may function by activating nociceptors, leading to pain through changes in neuronal and glial functions.^67^

### 3.4. Future directions for autoantibodies and their role in chronic pain

An outstanding question is how autoantibody production contributes to chronic pain that is regionally restricted. For instance, although some patients with CRPS develop pain that spreads widely from the site of injury, most cases experience pain localized to the injured limb. If autoantibodies are detectable in the serum, why are symptoms typically limited to one limb?^20^ One possibility is that autoantibody accumulation at self-antigenic targets in discrete tissues could recruit complement or produce localized inflammation in tissue niches, resulting in continued local deposition and restricted effects on nociceptors. The generation of autoreactive antibodies may also be regionally restricted. A recent study found that the mouse tibial fracture and cast model of CRPS results in lymph node enlargement and the emergence of germinal center B cells only in the popliteal lymph node ipsilateral to injury.^99^ This localized development of lymphocytes could account for the regionally restricted deposition of autoantibodies seen in this model, although autoreactive antibodies would likely be released into lymphatic circulation. In addition, the localized expression of self-antigens could support the induction and secretion of autoantibodies in discrete tissues. The subsequent formation of antigen-bound IgG-immune complexes may be sufficient to trigger complement activation or FcγR-mediated activity on nociceptors near the site of injury.

Further research is needed to explore the similarities and differences between IgG and IgM and their contributions to nociceptive hypersensitivity. Although there is evidence (presented below) that IgG autoantibodies exert some of their contributions to pain through interactions at FcγRs, there is considerably less known about how IgM can influence pain, although its effector functions are presumably due to complement recruitment or signaling at FcµRs. In 2 reports using the CRPS tibia fracture and casting model, IgM but not IgG protein was elevated in injured hind paw skin and sciatic nerve.^61,98^ Moreover, the authors found transfer of IgM, but not IgG, from either wild-type CRPS mice or patients with CRPS was pronociceptive to B-cell–lacking μMT mice,^61,62^ which differs from other reports on the pronociceptive role of CRPS IgG transfer.^67^ This contradiction may be due to the use of wild type vs B-cell–deficient μMT mice, as well as large differences in doses and duration of IgG transfer. Future research should investigate what brings about self-reactive IgG and/or IgM under conditions of pain.

## 4. Complement and its role in chronic pain

### 4.1. Complement: overview

The complement system supports host immunity by engaging a set of effector molecules to remove pathogens or damaged cells, amplify inflammation, and augment antibody responses.^33,129,168^ Complement is composed of more than 30 proteins found circulating in plasma and attached to cell surfaces. These complement elements are produced mainly by hepatocytes in the liver and by innate immune cells, including macrophages, monocytes, mast cells, and dendritic cells.^107^ There are several pathways to activation, but all result in a series of proteolytic cleavages of complement components, activating subsequent complement proteins in a well-characterized cascade. IgG and IgM antibodies are known to activate complement through the classical pathway, in which the C1 complex binds to the Fc region of antigen-bound antibodies.

### 4.2. Complement and pain

Elements of the complement system have been implicated in various pain conditions, including neuropathic pain, postoperative pain, and inflammatory pain.^39,137^ Early descriptive studies noted that elements of the complement system protein are elevated at the site of nerve injury, in the spinal cord dorsal horn and brainstem after peripheral nerve injury.^95,104,105^ Transcriptional analyses of spinal dorsal horn confirm that complement expression is induced after different models of nerve injury.^58,93^ Complement induction is functionally significant because either intrathecal or perisciatic delivery of soluble complement receptor 1 as a method to block C3a, C5a, and C5b-9 is sufficient to reverse mechanical allodynia from sciatic inflammatory neuropathy and chronic constriction injury.^170,171^ More specific pharmacological or genetic disruption of C5a or its receptor C5aR (including intrathecal and systemic delivery of C5aR antagonist PMX53) reduces cold allodynia and mechanical allodynia after partial nerve injury, reduces paw inflammation and pain after postsurgical paw incision, and attenuates inflammatory pain.^58,100,156^

Complement has been implicated in disease progression in arthritis; biomarkers of complement are elevated circulating in blood and in synovial tissues of patients with rheumatoid arthritis, and genetic deficiencies in receptors for C3a, C5a, or C6 protein all reduce disease severity in mice.^8,127,168^ However, it is not yet clear if the complement system contributes directly to joint pain. In the CAIA mouse model of rheumatoid arthritis, pharmacological antagonism of the C5a receptor did not block the development of pain-related behavior in response to CII autoantibodies, and complement 5 knockout (*C5*^−/−^) mice likewise develop pain-related behavior like wild-type mice.^12^ These results indicate, at least for the CAIA model, that pain hypersensitivity develops independently from C5.

In CRPS, it is also still unclear if complement contributes directly to pain, but there is circumstantial evidence linking autoantibody accumulation to pain. After tibia fracture and casting in the mouse model of CRPS, IgM accumulates in the injured hind paw skin and sciatic nerve, and there is also increased expression of complement C5b-9 membrane attack complexes at these sites.^98^ Depletion of B cells with anti-CD20 attenuates C5b-9 expression in the skin and nerve, implying that autoantibody release is a prerequisite for complement accumulation.^98^ Thus, binding of IgM bound to self-reactive targets at the site of traumatic injury and along primary afferents could lead to excessive complement recruitment, which could promote pain by damaging cells and peripheral nerves through membrane attack complexes or by elevating proinflammatory factors.^138^ CRPS is not typically associated with excessive tissue destruction, but localized damage to small-fiber peripheral nerves is consistent with the clinical presentation of CRPS. In patients with CRPS, a loss of intraepidermal nerve fibers has been observed in skin biopsies from the CRPS-affected limb compared with contralateral tissue,^126^ whereas other reports have found bilateral reductions in intraepidermal nerve fiber density in patients with CRPS compared with healthy controls.^142^ We speculate that the degeneration of nociceptive nerve fibers in affected tissue might be driven by complement. Cell lysis by membrane attack complexes could lead to focal damage at mixed peripheral sensory, motor, and autonomic nerves directly, or indirectly injure nerves by damaging vascular endothelial cells, keratinocytes, or other support cells.^124^ Although speculative, complement activation in CRPS may be restricted to autoantibody-bound targets in the periphery, accounting for localized tissue damage and inflammation.

### 4.3. Future directions for complement in chronic pain

Complement activation is important for coordinating host defense, but its recruitment may lead to detrimental amplification of pain under certain disease conditions. Recruitment of complement by autoantibodies could be pronociceptive through stimulation of proinflammatory cytokines locally in tissues, resulting in nociceptor activation.^156^ However, more clarity is needed to determine the downstream effects of complement activation that lead to pain. Additional research is also needed to determine if specific IgG or IgM autoantibodies in different disease models engage similar or separate elements of complement to produce nociceptor activation. Furthermore, during development in autoimmune demyelinating diseases, there is evidence that complement can tag neuronal synapses for microglial engulfment and elimination.^150,182^ Considering the importance of synaptic remodeling in the generation of chronic pain, there is a possibility that complement signaling to microglia could be an unexplored pathway for synaptic circuit reorganization contributing to pathological pain processing.

## 5. Fc gamma receptors and their role in chronic pain

Considering that the preponderance of research to date has explored the role of autoimmune IgG, this section will focus primarily on the role of FcγRs in chronic pain. However, there may be unexplored roles for other classes of Fc receptors that could be involved in pain or other sensory modalities. For example, expression of Fc epsilon receptor, the receptor for IgE, has been shown to co-localize to peripheral neurons and its activity may be important for allergic itch.^6,77,103^ FcµR, the receptor for IgM, may be expressed by oligodendrocytes, but it is not yet clear if it plays a role in nociception.^119^

### 5.1. Fc gamma receptors: overview

The classic surface-expressing FcγRs are transmembrane glycoproteins that recognize the Fc region of IgG. There are several classes of FcγRs that differ in their affinity for IgG isotypes, in their cellular localization, and in the downstream signal transduction pathways they engage.^69^ The FcγR classes can be functionally categorized as activating or inhibiting. Activating receptors promote phagocytosis, proinflammatory cytokine production, antibody-dependent cellular toxicity, oxidative burst, and cellular proliferation, whereas the downstream signaling of inhibiting receptors opposes these outcomes.^121^ There are several classes of activating FcγRs that differ between species, from humans (expressing FcγRI, FcγRIIA, FcγRIIC, FcγRIIIA, and FcγRIIIB) to mice (expressing FcγRI, FcγRIII, and FcγRIV), to rats (expressing FcγRI, FcγRIIA, and FcγRIIIA).^60,121,158^ In humans, mice, and rats, FcγRIIB is the sole inhibiting FcγR. For nearly all of these receptors, recognition of IgG occurs only when it has aggregated into IgG-immune complexes. Among the activating receptors, FcγRI is distinct in its high affinity for certain IgG isotypes, as well as its capability of binding both monomeric IgG along with IgG-immune complexes.

The role of FcγRs in regulating innate and adaptive immune responses in infection and autoimmunity has been extensively studied.^121^ For instance, the interactions between the complement system and FcγR signaling have long been studied for their role in rheumatoid arthritis pathogenesis.^94,157^ Our understanding of FcγRs has mostly derived from myeloid cells, although the functional outcomes they engage will naturally differ depending on the identity of the cell expressing them.^60^

Fc gamma receptors are expressed most prominently on immune cells, including macrophages, dendritic cells, natural killer cells, neutrophils, eosinophils, mast cells, and B cells. Beyond immune cells, there is evidence that cells within the central nervous system (CNS) also express FcγRs. Expression of FcγRI, FcγRII, and FcγRIII is observed in resected human brain tissue, although it is still unclear which cell types express these proteins.^172^ Microglia isolated from both humans and rats express FcγRs,^172,174^ and astrocytes from developing mouse and rat brains also have been characterized as expressing functional FcγRs.^122,158^ Importantly, the expression patterns of activating and inhibitory FcγRs on cells can change in states of injury, inflammation, and immune cell differentiation.^60^ Thus, in conditions of pathological pain, there may be dynamic alterations in FcγR expression among cell populations.

### 5.2. Fc gamma receptor expression on sensory neurons

In addition to their effect on immune cells, IgG may directly interact with FcγRs on sensory neurons to modulate nociceptive activity. This notion was derived from initial observations that primary sensory neurons from the mouse DRG express FcγRI as determined by immunostaining and Western blot.^5^ Several different approaches have confirmed this observation. Using immunofluorescence, it was verified that rat FcγRI is expressed at varying proportions on DRG cell bodies that co-express markers for peptidergic (CGRP or substance P) and nonpeptidergic (IB4) nociceptive neurons, and most small-diameter FcγRI^+^ DRG neurons express transient receptor potential V1.^136^ Transcriptional profiling of bulk samples indicates that mRNA of all 4 mouse FcγRs (*Fcgr1, Fcgr2b, Fcgr3,* and *Fcgr4*) are expressed in DRG.^12,144^ Labeling nociceptors with retrograde tracers reveals *Fcgr1* expression in ∼28% of joint-innervating neurons.^179^ Single-molecule fluorescent in situ hybridization further confirms expression of *Fcgr1* and *Fcgr2b* in mouse sensory neurons, and localization experiments suggest these transcripts are potentially transported along nerve fibers and accumulate at sites of nerve injury.^12^ Furthermore, immunoreactivity of mouse DRG cultures demonstrates co-localization of FcγRI and FcγRIIB with the neuronal marker βIII-tubulin, although their distribution differed: FcγRI was primarily localized to axons and neurites, whereas FcγRIIB immunoreactivity localized primarily on the cell bodies.^12^ Human DRG samples also express mRNA for all classes of FcγRs,^144^ although more evidence is needed to clarify the cellular and spatial distribution of FcγR protein, ie, whether it resides at DRG cell bodies, satellite glial cells, or primarily at neuronal projections in the periphery and axonal terminals in the spinal cord.

### 5.3. Fc gamma receptors and nociception

The expression of FcγRs on rodent DRG neurons is functionally relevant because application of IgG-immune complexes to activate neuronal FcγRs results in increased calcium influx, elevated neuronal excitability, and the release of substance P from cultured neurons.^5,75,136^ Elevated neuronal activity in response to FcγR activation may result in signaling through spleen tyrosine kinase to trigger opening of transient receptor potential canonical 3 ion channels (Fig. 1, center panel).^135^ This increased DRG neuronal activity is relevant for pain, as in vivo delivery of IgG-immune complex into the rat paw provokes mechanical allodynia and thermal hyperalgesia.^75^ Similar effects were seen in mice; localized injection of FcγR-stimulating bovine serum albumin IgG-immune complex, but not monomeric IgG, produces transient mechanical hypersensitivity of the ankle and glabrous skin without signs of tissue inflammation.^179^ Global genetic knockout of *Fcgr1* or conditional knockout of *Fcgr1* in *Pirt*^*+*^ sensory neurons reduces the increased pain-related behavior to IgG-immune complex treatment.^179^ Collectively, these results support the intriguing notion that antibodies could contribute to pain through their actions at FcγRs expressed on sensory neurons.

### 5.4. Fc gamma receptors and pain in rheumatoid arthritis

Autoantibodies can contribute to rheumatoid arthritis development through FcγR-mediated effector functions. Autoreactive IgG bound to self-antigens form immune complexes that can signal at FcγRs and disrupt the homeostatic balance between activating and inhibitory FcγRs, leading to improperly contained inflammation.^109^ Given that FcγRs are most abundantly expressed by immune cells, the contribution of FcγR signaling in the pathogenesis of rheumatoid arthritis was presumed to function through its effects on immune cells.^94^ Immunoglobulin G-immune complexes are known to bind FcγRs to activate neutrophils into releasing reactive oxygen species, proteases, cytokines, and chemokines, and presenting MHC class II antigens.^184^ Using the antigen-induced arthritis model (AIA), knockout mice lacking all 4 murine FcγRs show diminished bone erosion, and in wild-type mice, the extent of bone erosion positively correlates with the number of joint-infiltrating neutrophils.^31^ Bone erosion can be accelerated by neutrophil-derived tumor necrosis factor and IL-6 signaling to osteoclasts, but autoantibodies can also signal directly to FcγRs expressed by osteoclasts to further promote cell differentiation and joint destruction.^154^ Inhibiting FcγR signaling has shown promise for halting disease progression.^94^ Using the collagen-induced arthritis model in mice, injections of soluble recombinant human FcγRI to scavenge IgG-immune complexes reduced joint swelling and synovial tissue destruction.^35^ Thus, there remains a prominent role for FcγRs in mediating arthritis disease activity.^157^

Converging evidence from various animal models of rheumatoid arthritis has identified a role for FcγR-coupled signaling on sensory neurons as a mechanism for how autoantibodies could contribute to pathological pain.^12,75,102,179^ As previously mentioned, in the CAIA model of rheumatoid arthritis, injections of collagen-targeting anti-CII antibodies and LPS are sufficient to produce pain-related behavior before any signs of joint inflammation.^12^ Mechanical hypersensitivity to CAIA was also observed in mice lacking FcγRs on myeloid cells, but no pain-related behavior was seen if only hematopoietic cells expressed FcγRs, supporting the notion that pain-related behavior in this model arises from FcγR signaling on nonimmune cells.^12^ Cultured mouse DRG neurons show increased intracellular calcium signaling and release of CGRP neuropeptide in response to anti-CII-immune complexes, but not to monomeric CII antigen or antibody.^12^ These results align with in situ hybridization results that found around half of *Fcgr1*^*+*^ neurons co-expressed CGRP.^179^ Autoantibody immune complex–stimulated release of CGRP could signal at CGRP receptors on other neurons to cause nociceptor hyperexcitability. Ligand-receptor interaction analysis also suggests neuronal CGRP could influence the function of DRG glial cells (satellite glia and/or Schwann cells) by signaling at glial-expressed RAMP2.^180^ The outcome of CGRP treatment to satellite glial cells from trigeminal ganglia is the release of multiple proinflammatory cytokines, which can further exacerbate nociceptor activity.^1^ Based on these results, it is conceivable that autoantibodies, including ACPAs, accumulate at specific targets in the joint, forming immune complexes that can activate FcγRI on nociceptors at concentrations that do not evoke overt inflammation, producing arthralgia before edema and joint destruction.^12^

The role of neuronal FcγRI contributing to pain was confirmed using a separate model of arthritis (the AIA model). Both mice and rats show increased FcγRI expression in the DRG after AIA, particularly in CGPR^+^ neurons and small-diameter putative nociceptive neurons, and a greater proportion of joint-innervating sensory neurons were sensitive to IgG-immune complex stimulation after AIA induction.^75,102,179^ To investigate the functional role of neuronal FcγRI, conditional knockout mice and rats were generated in which *Fcgr1* is deleted from sensory neurons expressing the membrane protein phosphoinositide-interacting regulator of transient receptor potential (*Pirt*), offering a more selective cell-type–specific genetic perturbation.^102,179^ Accordingly, in both mice and rats, AIA-induced pain-related behaviors are alleviated when *Fcgr1* is deleted from *Pirt*^+^ sensory neurons.^102,179^ Likewise, pharmacological inhibition of FcγRI by delivering anti-FcγRI antibody (also known as anti-CD64) into the joint after AIA attenuates mechanical and thermal sensitivity with no effect on joint swelling.^179^ As previously mentioned, these results corroborate the notion that disease mechanisms driving joint pain may be partly uncoupled from joint destruction and inflammation.^81,91,159^

### 5.5. Future directions for Fc gamma receptors and their role in chronic pain

It remains an open question if FcγRs other than FcγRI are involved in pain. The repeated observation that somatosensory neurons express several classes of FcγRs raises the possibility that other FcγR classes promote nociceptive hypersensitivity similar to FcγRI. Given similarities in their structure and intracellular signaling pathways, there might be overlapping roles for activating FcγRs (FcγRIIA, FcγRIIC, FcγRIIIA, and FcγRIIIB in humans) to influence nociceptor hyperexcitability, or there may be loss of some unknown inhibitory function of FcγRIIB. Alternatively, there may be spatial restriction of FcγR expression within cellular compartments of neurons and glia, and their expression could involve dynamic trafficking in response to stimuli, as seen in response to injury with *Fcgr1* and *Fcgr2b* in mouse sensory neurons.^12^ It is unclear if this trafficking of *Fcgr* transcript in neurons is functionally relevant for pain. An intriguing report demonstrated expression of voltage-gated sodium channel Na_v_1.7 on dendrites of dorsal horn spinal neurons despite the lack of Na_v_1.7 mRNA and argues that mRNA or protein may be transsynaptically transferred from primary afferent sensory neurons to dorsal horn neurons.^4^ Although this may not be the case for FcγRs, these observations make it clear that it will be important to use multiple methods that can resolve cell-type expression (eg, single-cell RNA sequencing, in situ hybridization, and mass cytometry) to determine where FcγRs are expressed in DRG neurons, spinal cord, and brain. Fc gamma receptor expression has been identified on most immune cells as well as on somatosensory neurons, microglia, and astrocytes, but further characterization is needed to determine their cellular expression pattern in pain-relevant regions and how their expression may change in the maintenance of chronic pain.

## 6. Channelopathies from potassium channel complex autoimmunity

### 6.1. Potassium channel complex autoimmunity: overview

Activation of voltage-gated cation channels expressed by sensory neurons is a primary mechanism that determines nociceptor excitability to encode the detection of stimuli causing—or threatening to cause—tissue injury.^10^ VGKCs are expressed by neurons, including sensory neurons, and they function to hyperpolarize neuronal resting membrane potentials, thereby influencing action potential frequency and resultant neurotransmitter release.^54,141,169^ Proper coordination of VGKC activity with other ligand- and voltage-gated ion channels is therefore critical for normal sensory and motor function.^64^

Several neurological disorders have been described that are identified by the presence of VGKC complex autoantibodies (Box 3). These disorders are rare; from a sample of nearly 100,000 patients with suspected neurological autoimmunity, about 4% are positive for VGKC complex-IgG.^41^ Autoantibodies found in these disorders do not target the VGKC Kv1 subunits themselves, but rather most commonly bind to 3 proteins that complex with VGKCs within the cellular membrane: contactin-associated protein-like 2 (CASPR2), leucine-rich glioma inactivated 1, and contactin-2.^70^ Neuropathic pain as a symptom is particularly prevalent in patients with confirmed CASPR2-IgG, with the extremities being the most common location of reported pain.^79^ Contactin-associated protein-like 2 is a cell adhesion molecule in the neurexin family and is primarily localized at juxtaparanodal regions—sites of contact between myelinating glial cells and axons both in the CNS and at nodes of Ranvier in the peripheral nervous system—and in association with contactin-2 form complexes to cluster VGKCs at these regions.^132,133,167^ As such, autoantibody-mediated inhibition of VGKC complex expression or function can disrupt normal hyperpolarizing currents mediated by properly clustered potassium channels, thus leading to pain from increased neuronal excitability.

### 6.2. Potassium channel complex autoimmunity and pain

There is an increasing understanding of how VGKC complex antibodies are causative agents to pain hypersensitivity in these autoimmune neurological diseases. Neurons treated with VGKC complex antibodies show properties of hyperexcitability; IgG purified from patients with neuromyotonia causes repetitive firing of cultured mouse DRG neurons, and similar results are obtained when cells are treated with serum from CASPR2-IgG-positive patients, demonstrating the possibility of antibody interactions with DRG neurons.^29,155^ In this in vitro preparation, CASPR2-IgG treatment causes a reduction in membrane expression of Kv1.2 VGKCs, which mirrors the reduction in DRG surface VGKC expression seen in *Cntnap2*^−/−^ mice lacking CASPR2.^29^ The mechanism of how CASPR2-IgG reduces neuronal VGKC expression is likely not through internalization of CASPR2 but through binding CASPR2 and blocking its normal interactions with contactin-2, disrupting proper clustering of VGKCs on the axon at juxtaparanodal regions.^132^ Indeed, loss of *Cntnap2* is associated with exaggerated behavioral responses to mechanical or noxious chemical stimuli, which was accompanied by in vivo hyperexcitability of primary afferents and dorsal horn neurons in response to mechanical stimulation.^29^ Furthermore, similar to rheumatoid arthritis and CRPS, passive transfer of IgG from CASPR2-IgG-positive patients produces pain-related behavior in mice.^29^

Therapies of immunomodulation have shown some disease-modifying effects for pain. Treatment with different immunotherapies (such as prednisone, methylprednisolone, IVIg, methotrexate, or hydroxychloroquine) is frequently associated with pain improvement.^79^ In addition to rituximab, IVIg and plasma exchange were effective at improving symptoms for some patients with leucine-rich glioma inactivated 1–targeting antibodies.^71^

### 6.3. Future directions for potassium channel complex autoimmunity and pain

These results collectively indicate that autoantibodies can target potassium channel complexes on neurons producing heightened neuronal activity, thereby providing an explanation for the development of chronic pain in these neurological disorders. These autoantibodies presumably affect the firing rates of myelinated fibers; this would implicate activity from Aδ nociceptors, as well as the Aβ mechanoreceptors for innocuous touch whose dysfunction is implicated in mechanical allodynia.^28^ Future research should determine if different classes of somatosensory neurons are differentially affected by VGKC complex autoantibodies. Autoantibodies that are reactive with other neuronal antigens, such as antibodies targeting the NMDA glutamate receptor, are not necessarily associated with pain as a major symptom. However, autoantibodies for CNS targets have been implicated in psychiatric and neurological disorders. Antibodies targeting NMDA glutamate receptor can result in neurological deficits and psychosis; myasthenia gravis is a neuromuscular disorder caused primarily by autoantibodies against the acetylcholine receptor, and autoantibodies targeting receptors for the inhibitory neurotransmitter glycine have been implicated in encephalitis and stiff-person syndrome.^22,23,45,68^ This leaves open the possibility that autoantibodies reactive to neuronal or glial targets could influence pain sensation and perception.

## 7. Further considerations

The cumulative evidence reviewed here represents the remarkable progress made toward illuminating the ways in which autoimmune mechanisms contribute to pain in various disorders. Nevertheless, there are many questions related to autoimmunity and pain that remain to be answered. We highlight several of these questions here related to the development of autoimmunity, the influence of biological sex, the role of the brain in shaping immune function, and the unexplored role of autoantibody signaling at neurons and glia in the spinal cord and brain.

For conditions in which autoantibodies contribute to chronic pain, it is not clear what causes the initial shift from immune tolerance to autoimmune activation. This is a significant unanswered question with clinical implications for treating and preventing these conditions. There is still more research needed to identify what molecular triggers lead autoantigen-specific T cells and B cells to the production of autoantibodies during conditions of chronic pain. There may also be modifications to antibodies produced by B cells, such as glycosylation, that could alter the affinity of the Fab region to bind self-antigenic epitopes, or alter the Fc region to affect its ability to bind certain activating or inhibiting FcγRs, and/or change complement system recruitment. These modifications to self-IgG may shift “nonpathogenic” IgG into more disease-promoting autoantibodies.^151^ One could speculate that glycosylation of autoantibodies in specific tissue niches could shift the binding affinity to certain FcγRs expressed by neurons or immune cells.^121^

Biological sex is an important factor that should be considered in determining cellular and molecular explanations for autoimmune mechanisms of pain. Women have higher incidence of both chronic pain and likelihood of developing most autoimmune diseases.^36,110^ Given the vast differences in the probable risk factors and etiologies of different autoimmune diseases, there is unlikely to be a single biological pathway explaining these sex differences. However, there are potentially shared mechanisms associated with adaptive immune function. For instance, the autoimmune diseases systemic lupus erythematosus and Sjögren syndrome, which affect women at a rate 9 times higher than men, have the strongest genetic association with class II MHC human leukocyte antigen genes at the MHC locus. Variations in alleles for human leukocyte antigen genes have been associated with an increased risk for various autoimmune diseases.^145^ More recently, variations in complement genes *C4A* and *C4B*, found at the same MHC locus, strongly predict vulnerability for developing systemic lupus erythematosus and Sjögren syndrome in women, with women on average expressing lower protein expression of C4.^76^ Although further research is needed to elucidate the functional association between complement gene expression and autoimmune disease risk, reduced expression of complement could prolong adaptive immune cell interactions with self-antigens at sites of cellular injury or increase immune complex–mediated signaling at nociceptors.^76^

Considering the bidirectional signaling between the immune system and the nervous system, it is possible that neural circuits from the brain to immune targets could contribute to autoimmune function and thereby influence pain.^20^ Sympathetic innervation of primary and secondary lymphoid organs has been well characterized, and sympathectomy has been shown to influence the production of antibodies in the spleen.^108^ Immune cells across tissues have been shown to express neurotransmitter and neuroendocrine receptors, allowing for this neuronal control of immunity.^48^ To this end, a recent report identified a brain-to-spleen pathway controlling adaptive immunity, in which top-down activity from this neural circuit from central amygdala and paraventricular nucleus could directly influence the T-cell–dependent production of antibodies in the spleen.^189^ These observations hint at the exciting possibility that specific neural pathways could be targeted to more precisely influence antibody production. It has been thoroughly demonstrated that neural activity can regulate peripheral cytokine release and innate immune responses through vagal reflex circuits, and optogenetic activation of dopamine neurons in the brain has been shown to influence innate and adaptive immunity.^11,146^ Central mechanisms could conceivably influence autoimmune cellular function as well across tissues because neuronal release of CGRP can constrain cytokine production and proliferation of type 2 innate lymphoid cells in both the lung and the small intestine.^118,176,185^ Similarly, a newly described circuit of gut-innervating nociceptors can control localized immune responses to mediate host defense against pathogenic gut bacteria.^87^ These investigations highlight the sophisticated ways in which the CNS can influence immune function, and future challenges for treating pain arising from autoimmune mechanisms will involve advancing our knowledge of how the CNS can influence immune responses in health and disease.

Finally, most of the studies reviewed here focus on the role of autoimmune signaling at peripheral sensory input (in particular the DRG neurons) as contributing to pain. There is an established role of spinal cord neuroimmune signaling and plasticity of brain circuits as contributing to chronic pain, but the exact involvement of neural, glial, and immune cell signaling in spinal and brain circuits as contributing to autoimmune regulation of pain remains to be discovered.^74,83^ It will be important for future research concerning this topic to determine whether or not autoimmune mechanisms in the spinal cord and brain are initiated by changes in activity from peripheral input or if they are sustained independently from DRG activity.

## 8. Conclusion

Autoantibodies have been proposed as an important neuroimmune mechanism causing enhanced pain.^28,49,78,116,186^ In this review, we have focused on the sequence of events for how autoimmunity is established—from activation of B cells to autoantibody production, to the actions of autoantibodies recruiting complement, signaling at Fcγ receptors, and binding to self-reactive targets—and we have demonstrated how these autoimmune mechanisms can promote chronic pain across diverse conditions, including rheumatoid arthritis, CRPS, and autoimmune channelopathies. Beyond these chronic pain conditions, there is evidence in neuropathic pain that nerve injury upregulates FcγR expression in rodent spinal cord and DRG.^30,56,101^ This raises the possibility that autoantibodies and autoimmune signaling represent a generalized phenomenon across painful conditions, with different diseases involving distinct autoimmune mechanisms that contribute to the manifestation of chronic pain. Further elaboration of these autoimmune signaling properties and their contributions to pain could be critical for developing novel analgesic therapies.

## Disclosures

The authors have no conflicts of interest to declare.
